# Extracellular Matrix Patches for Endarterectomy Repair

**DOI:** 10.3389/fcvm.2021.631750

**Published:** 2021-02-11

**Authors:** Keith B. Allen, Joshua D. Adams, Stephen F. Badylak, H. Edward Garrett, Nicolas J. Mouawad, Steven W. Oweida, Manesh Parikshak, Parvez K. Sultan

**Affiliations:** ^1^St. Luke's Hospital of Kansas City, St. Luke's Mid America Heart Institute, Kansas City, MO, United States; ^2^Carilion Clinic Aortic and Endovascular Surgery, Roanoke, VA, United States; ^3^Department of Bioengineering, Department of Surgery, McGowan Institute for Regenerative Medicine, University of Pittsburgh, Pittsburgh, PA, United States; ^4^Cardiovascular Surgery Clinic, University of Tennessee, Memphis, Memphis, TN, United States; ^5^McLaren Bay Heart and Vascular, Bay City, MI, United States; ^6^Vascular Surgical Associates, Marietta, GA, United States; ^7^Cardiac Surgery Associates, Indianapolis, IN, United States; ^8^Cardio-Thoracic Surgeons, Birmingham, AL, United States

**Keywords:** endarterectomy, extracellular matrix, biologic patch, tissue regeneration, tissue integration, cerebrovascular disease, peripheral arterial disease, atherosclerosis

## Abstract

Patch repair is the preferred method for arteriotomy closure following femoral or carotid endarterectomy. Choosing among available patch options remains a clinical challenge, as current evidence suggests roughly comparable outcomes between autologous grafts and synthetic and biologic materials. Biologic patches have potential advantages over other materials, including reduced risk for infection, mitigation of an excessive foreign body response, and the potential to remodel into healthy, vascularized tissue. Here we review the use of decellularized extracellular matrix (ECM) for cardiovascular applications, particularly endarterectomy repair, and the capacity of these materials to remodel into native, site-appropriate tissues. Also presented are data from two post-market observational studies of patients undergoing iliofemoral and carotid endarterectomy patch repair as well as one histologic case report in a challenging iliofemoral endarterectomy repair, all with the use of small intestine submucosa (SIS)-ECM. In alignment with previously reported studies, high patency was maintained, and adverse event rates were comparable to previously reported rates of patch angioplasty. Histologic analysis from one case identified constructive remodeling of the SIS-ECM, consistent with the histologic characteristics of the endarterectomized vessel. These clinical and histologic results align with the biologic potential described in the academic ECM literature. To our knowledge, this is the first histologic demonstration of SIS-ECM remodeling into site-appropriate vascular tissues following endarterectomy. Together, these findings support the safety and efficacy of SIS-ECM for patch repair of femoral and carotid arteriotomy.

## Introduction

Arteries consist of an intimal lining surrounded by a thick, muscular media layer enveloped within the connective tissue of the adventitia (1). Together, these layers allow the artery to withstand high pressures from the heart (1,600–8,250 mmHg) (2). During the process of atherosclerosis, progressive accumulation of cholesterol, fatty acid salts, and tissue debris in the intima of the vessel wall leads to bulky atheroma formation and intimal tissue necrosis (3). Endarterectomy is a recommended approach to the management of significant atherosclerotic stenosis of the femoral or carotid arteries. In a surgical endarterectomy, an arteriotomy is performed and a cleavage plane is developed within the arterial wall between the intima and the media to remove the pathologic intima and to increase the luminal area of the artery (4). In some cases, the media may also be partially or completely removed during stripping of the atheroma, leaving the treated vessel without the smooth intimal lining and, possibly, portions of the muscular media (4–6).

Following endarterectomy, the arteriotomy can be closed by primary repair or with a vascular patch. Based on expanding evidence of superior outcomes with patch angioplasty compared to primary repair, patch closure is currently the preferred method (7–10). An ideal arterial patch must be able to withstand systemic arterial pressures over the long term, while maintaining low risk for restenosis, compliance similar to native artery, and resistance to thrombosis and/or infection (11, 12). ECM has also been investigated as a potential solution for replacement of small-caliber vessels. Some success has been reported using decellularized carotid artery ECM and human-engineered vessels as replacement small vessel grafts, and has been discussed in detail elsewhere (13, 14).

Vascular patches currently used for arterial reconstruction include autologous tissues and synthetic or biologic materials. Despite available evidence, choosing among these options remains a clinical challenge. Clinical studies have reported roughly comparable outcomes between autologous and non-autologous grafts, and between synthetic and biologic patches (7, 15, 16). The cost of synthetic patches are generally less than comparably-sized biologic patches, yet there are important differences between patch materials that may affect short- and long-term clinical outcomes. Autologous tissues have been widely used for endarterectomy repair, are readily accessed in most patients, and do not induce a foreign body response, but often require additional operative time, anesthesia, and result in increased patient morbidity due to the need for vein harvest (e.g., saphenous vein), and may affect future cardiovascular procedures (e.g., vein harvest for coronary artery bypass). Furthermore, vein grafts for arterial repair have demonstrated reduced compliance compared to native artery or even ECM-based biomaterials (17). Synthetic materials, such as polyethylene terephthalate (Dacron®) or polytetrafluoroethylene (PTFE), are ready to use and have a long shelf-life, have high biomechanical strength, but do not mimic the native vasculature, and stimulate a foreign body response on implantation which can lead to post-operative complications due to chronic inflammation, lack of remodeling, limited compliance, and poor resistance to infection.

Compared to autologous vein grafts, biologic materials [such as those derived from the extracellular matrix (ECM) of small intestine submucosa (SIS) and pericardium] have the advantage of off-the-shelf availability, obviating the need for a separate harvest procedure and its associated morbidity. Biological patches also have demonstrated advantages over synthetic materials, including a biologically natural three-dimensional (3D) structure, reduced risk for infection, good biocompatibility, an inflammatory profile that promotes healing, the presence of bioactive compounds (such as growth factors), and a natural capacity for conducting tissue turnover at the cellular level, through which these materials can be remodeled into native vascular tissues (17–20). The innate 3D structure of ECM includes natural cross-linking of collagen fibrils to adjacent fibrils to keep tissues strong and intact, yet allows for the ECM's natural degradation process to take place. Exogenous crosslinking of collagen fibers in ECM scaffolds can be performed during manufacturer processing with physical, biological, or chemical agents such as glutaraldehyde to enhance mechanical stability and minimize degradation of the ECM. However, exogenous crosslinking of ECM scaffolds may impede the natural remodeling process, resulting in calcification and potential scaffold failure (18, 21–23). Overall, the mechanical strength of synthetic materials exceeds that of non-chemically crosslinked biologic materials, in particular during the early stages of remodeling of the ECM into native tissues (24–26). However, non-chemically crosslinked biomaterials have demonstrated sufficient strength for vascular applications, including arterial repair (11, 17, 27). Importantly, biologic arterial patches made from SIS have demonstrated similar compliance to native arteries, and substantially greater compliance than autologous vein grafts or synthetic materials (17). It has been proposed that a biodegradable implant would be an ideal patch option if it supported the creation of organized, functional vascular tissues over time (1). Although biologic materials come with the disadvantage of lot-to-lot variability and potential uncontrolled bioactivity, thrombosis, and neointimal hyperplasia upon contact with blood, evidence from clinical and preclinical studies demonstrates that certain biologic patch materials display clinically-beneficial characteristics (15, 17, 18, 28–30).

Biologic patches composed of ECM are natural biomaterials that retain the properties and bioactive constituents of native tissue ECM. The decellularized ECM of non-chemically crosslinked biomaterials acts both as a scaffold and a stimulus for the ingrowth of new vascular tissue (17). These characteristics have led to the successful use of such biomaterials, including SIS-ECM, in a wide range of cardiovascular procedures, from repairs of congenital heart and vascular defects to vascular reconstructions following trauma, hemodialysis (arteriovenous fistulas), and surgical interventions (31–38).

This article discusses the regenerative characteristics of ECM and relates these properties to the benefits of ECM-based materials in endarterectomy patch repair. We present a discussion of observational data on the use of a SIS-ECM scaffold for iliofemoral and carotid endarterectomy reconstruction (previously presented in part) (39) and a histological analysis of SIS-ECM explanted from a previous endarterectomy repair, which demonstrates the regenerative potential of this biologic material. Finally, we compare these findings to previously published endarterectomy patch experiences with SIS-ECM scaffold used for the same application (31, 32, 40–42).

## Benefits of ECM-Based Biomaterials: Bioactivity, Infection Resistance, and Constructive Remodeling

Based on current evidence, ECM-based materials may be preferred to other options for endarterectomy patch repair. As noted, clinical outcomes with biologic, synthetic, and autologous grafts appear to be roughly comparable. However, ECM-based materials have numerous potential advantages as outlined below.

### ECM Materials Are Bioactive

The structure of ECM transmits biomechanical forces and serves as a substrate for cell migration and differentiation. Because it is biologically derived, this structure can adapt following implantation, providing not only structural support, but also a range of bioactive compounds. Components of ECM are highly conserved across species and include collagens, glycoproteins, proteoglycans, mucins, elastin fibers, and growth factors, each of which may influence the function of the ECM, host response, and remodeling (20). In addition to containing a range of structural and functional proteins, non-chemically crosslinked ECM materials are naturally degraded by proteases, a process that releases additional bioactive peptides and degradation products. ECM degradation is a natural activity present in all living tissues and is paramount to tissue remodeling.

The rate of ECM scaffold degradation varies across material types. For example, degradation of ECM is inhibited by chemical crosslinking, whereas certain methods of terminal sterilization may increase degradation rate (43–45). Non-chemically crosslinked ECM materials are typically degraded steadily after implantation. Approximately 60% of ECM mass is degraded and resorbed within 4 weeks of implantation, with full degradation by ~3 months (46, 47). The rate of ECM scaffold degradation appears to be similar to the rate of new tissue deposition, although there can be a loss of mechanical strength before the degraded ECM is fully replaced by host tissues (45). Peripheral blood-derived macrophages are central to the early and rapid degradation of ECM scaffolds (45).

In addition to releasing naturally occurring anti-microbial peptides (AMPs), this degradation process exposes molecular sites and signaling molecules that influence cell behaviors such as chemotaxis, adhesion, differentiation, and angiogenesis (20, 48–54) [For an extensive review on matricellular proteins, see Ramaswamy et al. (55)] In other words, degradation of ECM-based materials by the host appears to be central to the successful regeneration of native tissues. This bidirectional interaction between cells and the ECM has been termed dynamic reciprocity (20).

### ECM Materials Are Resistant to Infection

The *in-vivo* antimicrobial effects of ECM-based bioscaffolds are not the direct result of inherent ECM molecular structure or composition. When intact SIS is exposed to bacteria *in vitro*, microbial growth is not inhibited, which contrasts with findings in preclinical animal models (56). However, when ECM materials are digested/degraded *in vitro*, the resulting degradation products show robust antibacterial activity (49, 57). In fact, the degradation of ECM materials *in vivo* is critical to their demonstrated ability to resist infection and remodel into native tissues. This *in vivo* degradation process explains the discrepancy between animal and *in vitro* studies, in which the ECM is not digested by acids or proteases *in vitro* and therefore does not release cryptic antimicrobial peptides (AMPs) and other bioactive factors.

Synthetic materials can harbor bacteria and generally must be removed when infected (28, 29). In a porcine model comparing SIS-ECM to expanded PTFE (ePTFE), vascular grafts were implanted and deliberately contaminated with *Staphylococcus aureus* or *S. epidermidis* (30). During the 6-week study period, inoculated SIS-ECM grafts demonstrated less neointimal hyperplasia, extensive remodeling into native arterial tissue, and importantly, no bacterial growth compared to ePTFE grafts, which had high bacterial counts. Another porcine study comparing SIS-ECM to PTFE for vascular reconstruction in the setting of gastrointestinal contamination reported a 73% rate of infection in the PTFE group, compared to 8% in the SIS-ECM group [*P* < 0.03; (58)]. The rate of pseudoaneurysm was also lower with SIS-ECM (25% PTFE vs. 0% SIS-ECM). Studies in other animal models have reported similar findings: that is, resistance to bacterial infection with biological materials, such as SIS-ECM, compared to inflammation and bacterial growth with ePTFE or Dacron (59, 60).

### ECM Materials Are Immunomodulatory and Support Constructive Remodeling

The host response to ECM-based materials, especially those of xenogeneic (usually porcine) origin has been extensively investigated (18, 61–64). The processing of ECM-based materials removes not only cells but also cell-associated xenogeneic epitopes [e.g., Galα1,3Galβ1,4GlcNAc-R (α-Gal)] responsible for antigenicity, thereby minimizing the risk for immune rejection. Decellularized, non-chemically crosslinked ECM-based materials have been shown to modulate the native immune response to favor constructive remodeling, while mitigating chronic inflammation (64). Part of the complex cellular response to an implanted prosthesis such as ECM is the attraction and polarization of macrophages. Two broad phenotypes of macrophages, often called M1 and M2, promote differing responses to the prosthesis. Macrophages with a dominantly M1 phenotype promote inflammation and the killing of pathogens, whereas macrophages with a dominantly M2 anti-inflammatory phenotype promote immunoregulation and constructive remodeling of tissues (18, 21). Studies have demonstrated that ECM-based materials that are not extensively crosslinked stimulate a response characterized by the presence of M2 macrophages, which are associated with incorporation into native tissue, whereas extensively crosslinked ECM and synthetic materials encourage an M1 phenotype, which is associated with chronic inflammation, resistance to degradation, and a foreign body response (18, 21–23, 26, 64).

The early transition from M1 proinflammatory to M2 pro-remodeling macrophage response is a necessary step toward constructive remodeling of ECM-based materials and a favorable clinical outcome (45). In fact, the early macrophage phenotypic response to ECM implants can predict subsequent remodeling outcomes (65). The specific mechanisms by which ECM-based materials modulate this phenotypic response are not fully understood, but studies report that it is linked to the release of bioactive peptides, proteins, and matrix bound nanovesicles (MBV) from the degrading ECM (45, 66–68). The ability to recruit and control differentiation of stem and progenitor cells through the release of these bioactive peptides is what drives ECM-mediated tissue remodeling (69). Other factors that contribute to constructive remodeling include the application of site-appropriate mechanical loading, as is generated following implantation of ECM-based materials into living tissues, such as arteries (70, 71). The remodeling of SIS-ECM has been demonstrated clinically as early as 3 months post-implantation and, in animal studies, as early as 2 weeks, with complete remodeling within 6 months (27, 30, 72–75).

When considering biologic grafts, the manufacturing processes used to create specific materials must also be considered. The importance of patch manufacturing processes is demonstrated by the impact of chemical crosslinking on macrophage phenotype, as discussed above. Extensive chemical crosslinking and certain methods of decellularization, sterilization, and other manufacturing processes can alter the native ECM architecture, physiochemical properties, and growth factor content, potentially attenuating constructive remodeling of the patch and instead promoting inflammation, foreign body response, and encapsulation (17, 20, 44, 45, 65). Unlike SIS-ECM, other currently available vascular patch products, which are either synthetic or made from chemically crosslinked animal tissues (such as bovine pericardium), have been shown to elicit a sustained M1 macrophage phenotype and a foreign body response characterized by scarring, calcification, and fibrosis (17, 22, 26, 76, 77).

## Clinical Experience With ECM as an Endarterectomy Patch

### SIS-ECM in Iliofemoral and Carotid Endarterectomy: Observational Data

Despite growing evidence of the utility of biologic patches in vascular surgery, the clinical performance of individual materials remains incompletely described. An ECM patch derived from porcine SIS (SIS-ECM) is commercially available, constructed of multi-laminate (six-ply), decellularized, non-chemically crosslinked, lyophilized, and specifically indicated for use as a patch material in vascular reconstruction (VasCure®, Aziyo Biologics, Silver Spring, MD, USA). All investigators for the data reported herein prepared the patch according to manufacturer instructions for use (hydration in sterile isotonic solution for 1–2 min prior to implantation).

The safety and efficacy of this SIS-ECM for vascular repair following endarterectomy was evaluated in two manufacturer-sponsored, prospective, observational, post-market studies of 259 patients undergoing iliofemoral repair (the PERFORM Study) and carotid patch repair (the Carotid Registry). These studies were conducted with the approval of each site's IRB or through a centralized IRB. All enrolled subjects were consented with their site's approved Informed Consent Form. Across datasets, the repairs achieved high procedural success rates (~100%) and high patency up to 12–24 months of follow up, with very low rates of restenosis (<3%), and adverse event rates comparable to those reported by other studies of patch angioplasty. Of the reported adverse events, <0.8% were reported as definitely related to the SIS-ECM patch. These results support the safety and efficacy of this SIS-ECM material for the repair of iliofemoral and carotid endarterectomies.

Also presented is a histologic analysis of explanted SIS-ECM from one patient after a healed femoral endarterectomy repair and obtaining informed patient consent. We believe this is the first histologic report of site-appropriate vascular tissue remodeling of SIS-ECM following endarterectomy.

### SIS-ECM in Iliofemoral Artery Reconstruction—PERFORM Study

Data from 38 symptomatic patients (45 femoral arterial reconstructions) with peripheral arterial disease (PAD) undergoing iliofemoral endarterectomy and arteriotomy closure with SIS-ECM patch repair were collected at three centers. Patient demographics, comorbidities, indications for endarterectomy, procedures performed, and follow up time are listed in Table 1.

**Table 1:** Patient demographics, comorbidities, indications, and procedures—PERFORM study.

**Characteristic**	**Value**
Number of patients (*n*)	38
Mean age, years ± SD	63.7 ± 11.0
Sex, male, *n* (%)	27 (71.1)
**Race**, ***n*** **(%)**	
White	29 (76.3)
Black or African American	9 (23.7)
**Comorbidities**, ***n*** **(%)**	
Hypertension	35 (92.1)
Hyperlipidemia	26 (68.4)
Diabetes	18 (47.4)
Obesity	3 (7.9)
Active tobacco use	15 (39.5)
Sedentary lifestyle	12 (31.6)
Family history of vascular disease	12 (31.6)
Previous vascular surgery	20 (52.6)
Previous groin surgery	13 (34.2)
Previous myocardial infarction	9 (23.7)
History of coronary artery bypass grafting	10 (26.3)
Chronic renal insufficiency	7 (18.4)
**Indications for endarterectomy**, ***n*** **(%)**	
Claudication	34 (79.1)
Acute critical limb ischemia	7 (16.3)
Rest and night pain	4 (9.3)
Gangrene	2 (4.7)
Other	2 (4.7)
**Procedure performed**, ***n*** **(%)**	
Iliofemoral endarterectomy	30 (66.7)
Femoral endarterectomy	11 (24.4)
Iliofemoral endarterectomy with profundaplasty	3 (6.7)
Other	1 (2.2)
**Patient follow up**	
Days of hospital stay ± SD (range)	5.5 ± 4.6 (1–18)
Days of follow up ± SD (range)	252 ± 166 (29–448)
Patients completing 12-month follow-up, *n* (%)	21 (55.3)
Procedures completing 12-month follow-up, *n* (%)	24 (53.3)

The procedural success rate was 100%. Patency was measured by duplex ultrasound and patient-reported complications, and was maintained in 42 out of 43 arteries (97.7%) of procedures through a mean follow-up of 252 ± 166 days (range 29–448). The only patient with a non-patent limb had Fontaine Class III PAD and underwent above-the-knee left leg amputation due to failed repeat revascularization procedures. Over 12 months, there were zero (0%) adverse events reported that were considered related to the SIS-ECM patch. Importantly, there were no (0%) patch ruptures, explants, patch-related pseudoaneurysms, or patch infections.

Five patients (13.2%) experienced a total of seven procedure-related adverse events (Table 2), which is consistent with previously reported rates in the literature (78–80). One pseudoaneurysm was reported 3 days after the index procedure due to a broken anastomosis suture; the ECM patch and femoral artery remained intact in this patient. A superficial wound infection was reported for one patient, and seroma was reported for two patients, one of which was infected; both were successfully treated without explantation of the SIS-ECM patch. The incidence of adverse events did not differ between patients with or without previous groin surgery (*P* > 0.05).

**Table 2:** Procedure-related adverse events—PERFORM study.

**Adverse event**	***n* (%)**
Pseudoaneurysm (suture break)	1 (2.3)
Superficial site seroma	2 (4.7)
Superficial wound infection	1 (2.3)
Pain and/or numbness of extremity	3 (7.0)

These efficacy findings align with published studies of femoral endarterectomy with patch angioplasty, which have reported 1-year and long-term patency rates of 90–100% and 85–96%, respectively (78–82). However, limited prospective evidence has been published comparing outcomes of SIS-ECM with different types of patches for femoral endarterectomy.

### SIS-ECM for Carotid Artery Reconstruction—Carotid Registry

Data from 221 patients undergoing standard interventional carotid endarterectomy and SIS-ECM patch repair were collected at six centers. The demographic characteristics, comorbidities, and procedures performed are listed in Table 3. Follow-up evaluations, including carotid duplex imaging, were performed at 1–3, 6, 12, and 24 months after the index procedure. A follow-up evaluation was considered completed only if duplex imaging was available for the subject/visit.

**Table 3:** Patient demographics, comorbidities, and procedures—Carotid Registry.

**Characteristic**	**Value**
Number of patients (*n*)	221
Number of patients with preoperative stenosis >50% (%) (per duplex ultrasound or CT)	219 (99.1)
Mean age, years ± SD	69.9 ± 10.0
Sex, male, *n* (%)	118 (53.4)
**Race**, ***n*** **(%)**	
White	203 (91.9)
Black or African American	16 (7.2)
Asian	2 (0.9)
**Comorbidities**, ***n*** **(%)**	
Hypertension	182 (82.4)
Diabetes	84 (38.0)
Obesity	47 (21.3)
Active tobacco use	75 (33.9)
COPD	32 (14.5)
Previous neck surgery	34 (15.4)
Previous neck radiation	2 (0.9)
Previous transient ischemic attack	51 (23.1)
Previous transient ischemic attack (symptomatic)	22 (10.0)
Previous stroke	43 (19.5)
Previous stroke (symptomatic)	20 (9.0)
Valve disease	9 (4.1)
Previous atrial fibrillation	22 (10.0)
Previous myocardial infarction	45 (20.4)
Congestive heart failure	19 (8.6)
Chronic renal insufficiency	19 (8.6)
**Procedure performed**, ***n*** **(%)**	
Internal carotid endarterectomy	220 (99.5)
Common carotid endarterectomy	1 (0.5)

The results show reductions in stenosis following carotid endarterectomy (Table 4), with mean change from baseline in maximum carotid stenosis of at least 50% across all time points with patency maintained in 89% of patients through 24 months. Restenosis occurred in six (2.7%) subjects. These findings align with previous studies, which reported long-term patency rates of 79–100% with synthetic patches and 87–100% with biologic materials following carotid endarterectomy (7, 11, 83). There was a low rate of adverse events reported: 10 (4.5%) were reported as possibly related to the device, 1 (0.45%) was reported as probably related to the device, and 2 (0.9%) (occurring in the same patient) were reported as definitely related to the device (Table 5).

**Table 4:** Follow-up of stenosis in subjects who underwent carotid repair.

**Time point (months)**	**Completed follow-up *n* (%)**	**Restenosis <50% *n* (%)**	**Maximum mean carotid stenosis, %**	**Mean change from baseline in carotid stenosis**	
				**Maximum, %**	**Minimum, %**
Baseline	NA	NA	85.5	NA	NA
1–3	198 (89.6)	26 (13.1)	32.2	−52.5	−64.5
6	167 (75.6)	26 (15.6)	32.6	−52.6	−63.9
12	166 (75.1)	24 (14.5)	33.6	−51.8	−63.4
24	155 (70.1)	17 (11.0)	33.8	−51.1	−64.2

**Table 5:** Adverse events possibly, probably, or definitely related to the SIS-ECM patch—Carotid Registry.

**Adverse event**	***n* (%)**
**Possibly related**	
Restenosis	6 (2.7)
Irregular area of left bifurcation/distal common carotid artery patch	1 (0.45)
Occlusion	1 (0.45)
Thrombus on wall of carotid artery opposite ECM patch	1 (0.45)
Hematoma	1 (0.45)
**Probably related**	
Bleeding status post-left carotid endarterectomy	1 (0.45)
**Definitely related**	
Patch dehiscence/patch rupture	1 (0.45)
Pseudoaneurysm	1 (0.45)

### Histology Reveals Transformation of SIS-ECM at Femoral Artery Endarterectomy Site

**Figure 1:**
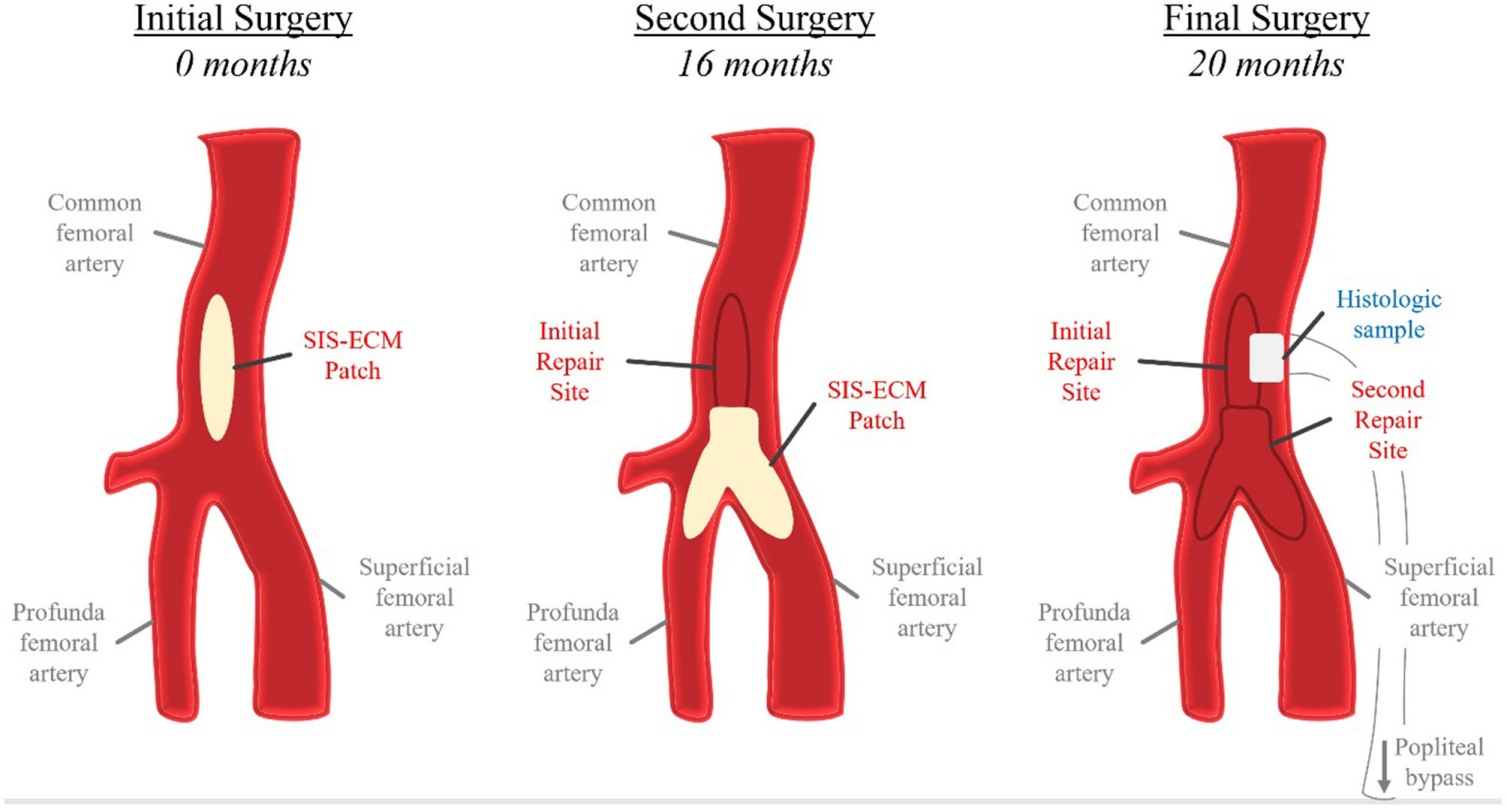
Schematic representation of repeated iliofemoral endarterectomies in the histologic case study. At the initial surgery, a SIS-ECM patch was placed in the common femoral artery (CFA) following removal of the atheroma. The second surgery was conducted 16 months later due to progression of disease distal to the previous SIS-ECM repair. Following endarterectomy, a Y-shaped SIS-ECM patch was placed at the branching of the CFA, profunda femoral artery, and superficial femoral artery. The previous SIS-ECM patch remained widely patent. The final surgery, a femoral-to-above-knee popliteal artery bypass, was conducted at 20 months after the index procedure due to progression of distal disease. Both previously placed SIS-ECM patches remained widely patent. At this time, a biopsy of the original SIS-ECM patch, including the area of anastomosis between the patch and the native artery, was obtained for histologic analysis.

**Figure 2:**
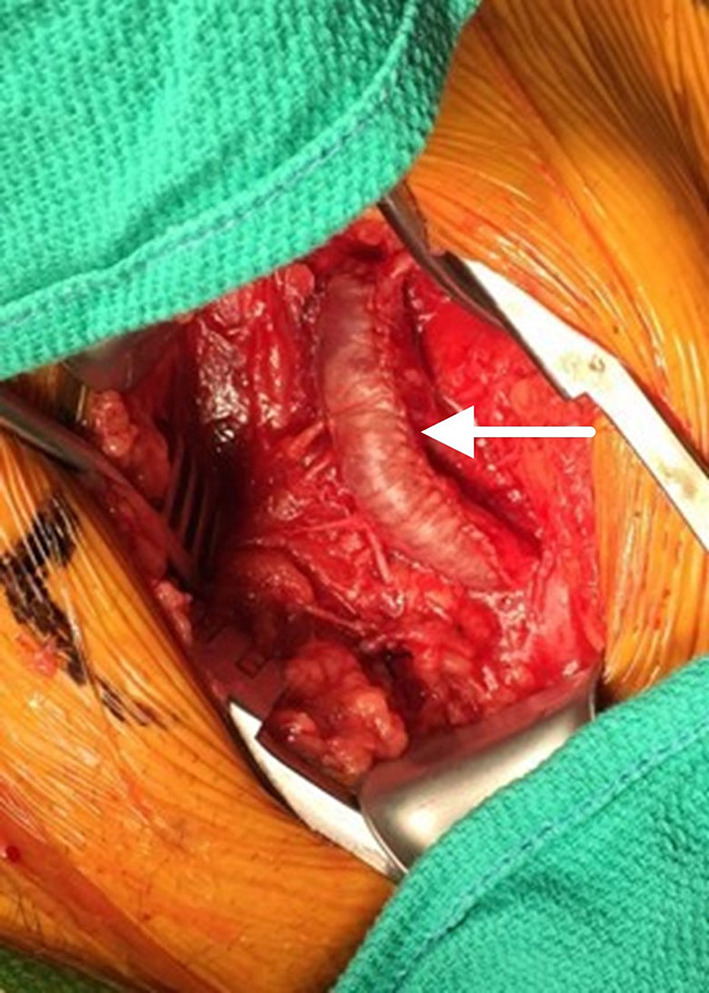
Initial patch angioplasty of the common femoral artery using an SIS-ECM patch (arrow) following endarterectomy.

**Figure 3:**
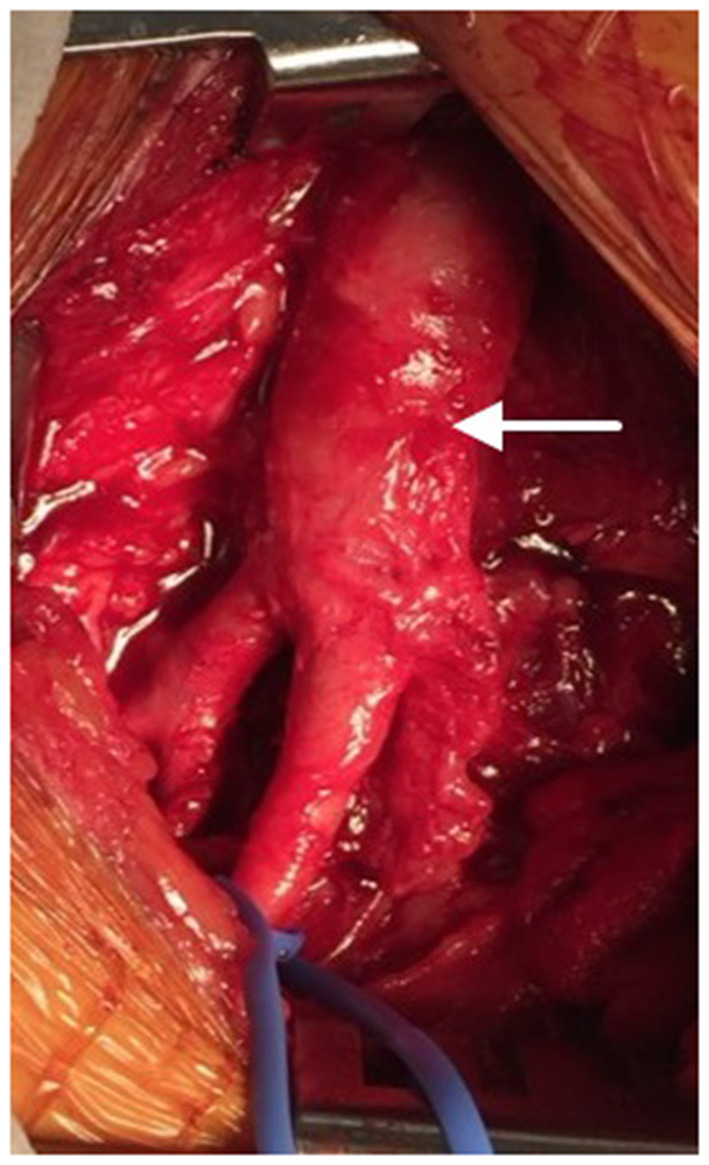
After 16 months, the SIS-ECM patch previously placed in the right common femoral artery (CFA) (arrow) was found to be completely incorporated into native vascular tissue.

The regenerative potential of SIS-ECM when used in patch angioplasty is demonstrated through histology from a complicated case showing successful integration of the SIS-ECM patch into site-appropriate endarterectomized vessel tissue. A 55-year-old Caucasian male underwent right CFA endarterectomy with patch angioplasty using a SIS-ECM patch (Figures 1, 2) with no subsequent adverse events. Sixteen months later, due to disease progression at a distal adjacent site, an additional operation was performed. The previously placed SIS-ECM patch in the right CFA was found to be grossly indistinguishable from the surrounding endarterectomy site arterial tissue. At this surgery, the patch could be identified only by the presence of the polypropylene sutures used for the initial repair (Figures 1, 3). An additional endarterectomy and vascular reconstruction were performed with another SIS-ECM patch, extending the previous patch. On follow-up, the patient did well, with unremarkable non-invasive testing.

**Figure 4:**
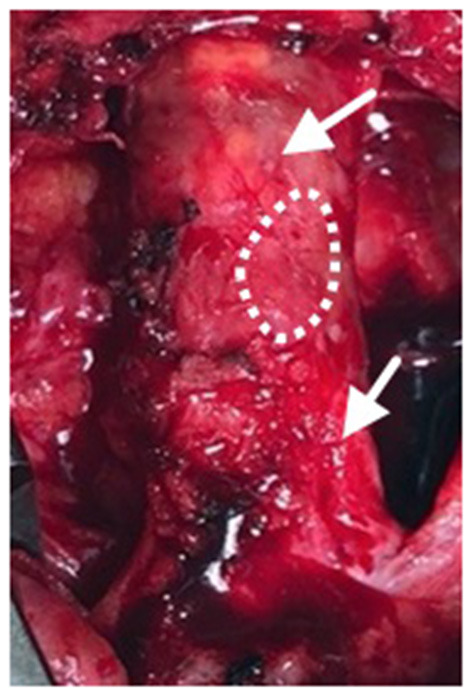
Following progression of distal disease 20 months after the index procedure, a third operation was performed with creation of a right femoral to above-knee popliteal artery bypass. At this time, the two previous SIS-ECM patches in the common femoral artery appeared to be completely incorporated into the native femoral system (arrows). A specimen, including the anastomosis between the patch and the endarterectomized artery, was removed for histologic evaluation (dashed outline).

**Figure 5:**
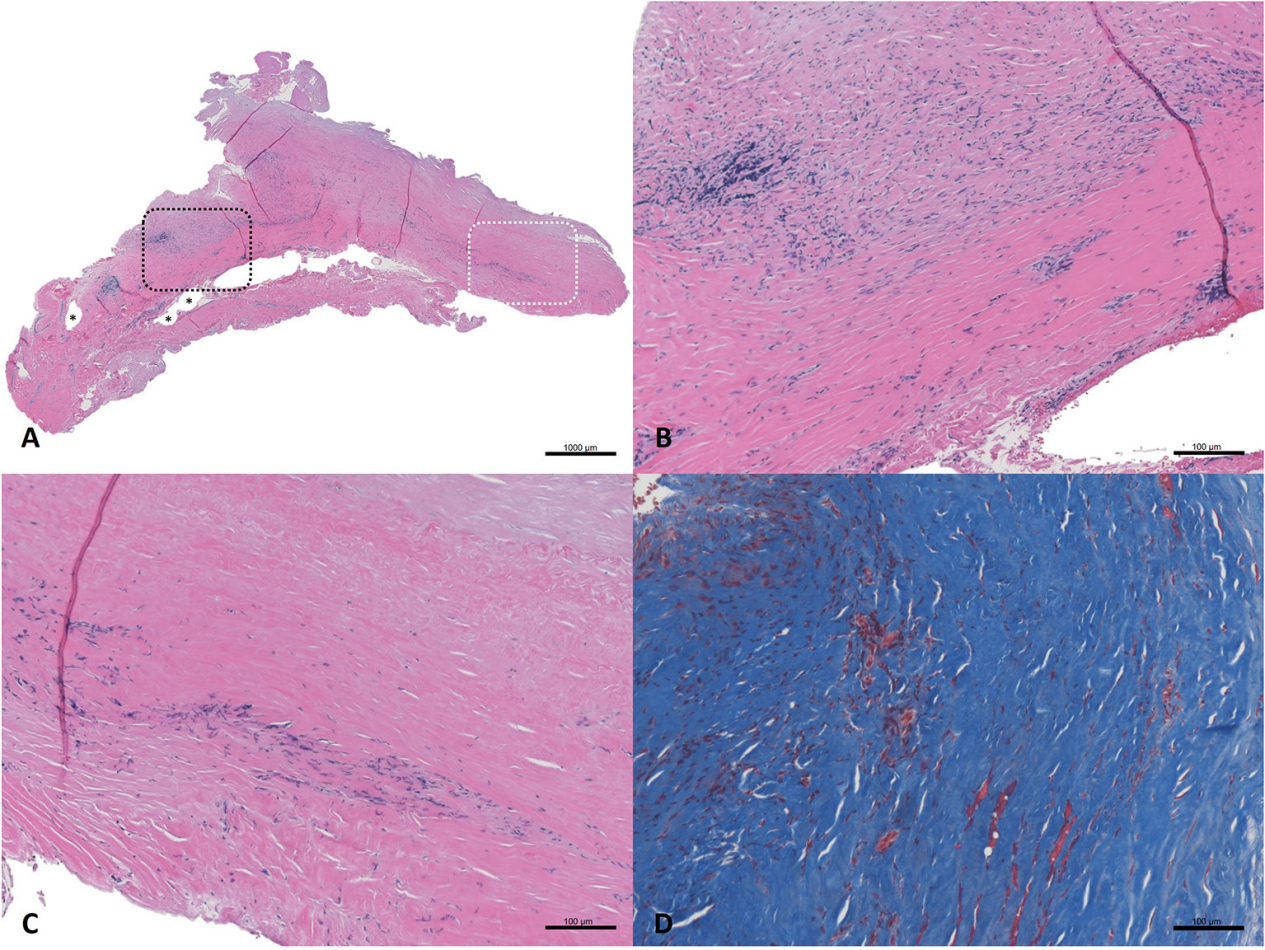
Haematoxylin and eosin-stained sections **(A–C)** and Masson's trichrome stained section **(D)** from SIS-ECM graft explanted from the common femoral artery. **(A)** Full field of view; a black dashed box marks an area of remnant graft material, and asterisks (*) denote intact suture holes. An area of interest is highlighted with a white dashed box which represents remodeled graft. **(B)** Detailed view of a full thickness section of remnant graft material undergoing active remodeling (top of the image), surrounded by organized collagen (bottom of the image). **(C)** Detailed view of the area of interest; a full thickness section of the remodeled graft, with no evidence of the originally implanted ECM material remaining. The more luminal side of the graft (top of the image) shows well organized and aligned collagen, minimal cellularity, and no evidence of inflammation. The more abluminal surface (bottom of the image) shows slightly less organized collagenous connective tissue, moderate, and localized cellularity consistent with residual remodeling, and no evidence of active inflammation or necrosis. The collagenous tissue shows a population of morphologically normal spindle cells, which are most likely fibroblasts. **(D)** Detailed view of trichrome stained tissue representing a remodeled ECM area showing well organized, dense accumulation of collagen. The tissue was aligned in a circumferential fashion, as would be expected for a normal blood vessel. Small areas of red staining represent islets of smooth muscle.

Nineteen months after the initial procedure, the patient again developed disabling symptoms and presented with further progression of distal disease. A month later, a right femoral-to-above-knee popliteal artery bypass was performed using a prosthetic conduit. At the time of this surgery, both previously placed SIS-ECM patches were indistinguishable from the surrounding vascular tissue and could only be identified by the perimeter polypropylene sutures used to implant the patches (Figures 1, 4). Similar to the fate of the first patch used, the second patch was also visually indistinguishable from the rest of the vessel walls. Since this procedure required a longitudinal arteriotomy in the CFA at the level of the initial SIS-ECM patch, a tissue specimen including the anastomosis between the first patch and the previous endarterectomized vessel was removed and sent for evaluation by an independent laboratory. Analysis of the specimen showed the area of the patch to be histologically identical to the adjacent endarterectomized vascular tissue, with no evidence of inflammation or degeneration in any areas of the patch (Figure 5). Post-operatively, the patient had normal non-invasive testing and complete resolution of their lower extremity discomfort.

## Discussion

### Efficacy of SIS-ECM Patch Following Endarterectomy

Overall, studies comparing synthetic and biologic patch materials for endarterectomy repair have reported similar outcomes (e.g., patency rates, durability, risk for adverse events such as pseudoaneurysm, and survivability) across patch types (16, 83). The majority of current evidence for biologic patches relates to the use of bovine pericardium in carotid endarterectomy. For example, a 2018 meta-analysis of 18 randomized trials comparing bovine pericardium to synthetic patch materials (Dacron or PTFE) or vein grafts for repair of carotid endarterectomy found no significant differences between groups across a range of outcomes (15). When comparing bovine pericardium to synthetic patch materials, no significant differences were found in the incidence of 30-day stroke, transient ischemic attack, local neck hematoma, or death.

More recently, an analysis of primary carotid endarterectomy cases recorded in the Vascular Quality Initiative (VQI) registry compared outcomes between bovine pericardium, autogenous vein, Dacron, and PTFE patches (7). At 1-year post-procedure, bovine pericardium had the lowest incidence of restenosis and was also associated with a lower incidence of return to the operating room. Bovine pericardium and Dacron had lower incidence of stroke or transient ischemic attack compared to PTFE, vein patch, or primary repair. The authors concluded that this large dataset enabled identification of superior outcomes with bovine pericardium post-operatively and at 1 year compared to synthetic and autologous patch materials.

With regard to SIS-ECM specifically, a published case study of a patient undergoing repeat carotid endarterectomy used the same SIS-ECM patch used in the present studies (31). The authors reported procedural success with the SIS-ECM material and high patency of the patched internal carotid during follow up. However, no histologic analysis was reported evaluating the tissue characteristics of this successful use.

A recent case series of four patients undergoing femoral endarterectomy reported experience with the same SIS-ECM material used in this study (32). One patient died of cardiac arrest 2 days after the procedure; the other three showed widely patent common femoral arteries at the area of the patch repair 1 year post-procedure. Although the authors reported overall clinical success when using the ECM patch for this application, no histologic analysis was reported to characterize the tissue.

The results reported here not only add to a growing evidence base supporting the efficacy of SIS-ECM for vascular reconstruction, but the incorporation of the SIS-ECM into endarterectomized vascular tissue is illustrated by the histology described above and provides a specific example of the fate of SIS-ECM when used in this application. To our knowledge, this is the first histologic example showing that SIS-ECM functionally adopts the anatomy of adjacent endarterectomized vascular tissue. In this complex patient, histologic analysis of the patch sample obtained upon reoperation demonstrated moderately dense, well-organized collagen, and occasional capillaries (Figure 5). While small areas of remnant patch material could be identified at 20 months post-implantation, even these scattered regions were characterized by remodeling and replacement with organized collagen. Importantly, no evidence of inflammation was detected.

### Safety of SIS-ECM Patch Following Endarterectomy

Although generally safe, endarterectomy with patch angioplasty has been associated with rare but serious complications, such as patch rupture, patch infection, and pseudoaneurysm formation. No (0%) adverse events were reported in the histologic case study related to the SIS-ECM patch. In the PERFORM dataset, there were also no (0%) device-related adverse events, which compares favorably to rates reported in other studies evaluating venous, bovine, and prosthetic graft materials following endarterectomy (84–86). Only one patient (2.3%) developed a pseudoaneurysm, and investigation determined the cause to be a broken suture along the line of the anastomosis, with the ECM patch remaining intact.

The Carotid Registry experience identified one case of pseudoaneurysm related to the patch, one case of patch dehiscence/rupture, and no cases of patch infection. This low rate of pseudoaneurysm (0.45%) compares favorably with rates of 0.2–3.6% reported in studies of other patch materials (10, 16). Resistance of SIS-ECM to rupture and pseudoaneurysm demonstrated by these data may relate to the greater thickness of the six-ply SIS-ECM compared to other patches and the ability of SIS-ECM to conform to the repaired vessel and remodel into native tissue.

Few previous case series have reported higher rates of pseudoaneurysm with SIS-ECM (40–42). One study of a four-ply SIS-ECM material (Cook Biotech Inc., West Lafayette, IN) for patch closure of carotid endarterectomy reported no adverse events with the first 69 of 76 patients implanted with the patch (40). Subsequently, seven patients (9.2%) developed asymptomatic pseudoaneurysms, six of which required surgical intervention. Mechanical testing of patches from the same material lots associated with pseudoaneurysm identified thinner and more variable physical characteristics compared to a control lot. Of important note, the SIS-ECM patch used in the endarterectomy studies reported here consists of six layers (VasCure®, Aziyo Biologics, Silver Spring, MD, USA), and is therefore thicker and stronger than the SIS-ECM that was used in the referenced study.

A case series using this same six-ply SIS-ECM reported three cases (8.1%) of pseudoaneurysm out of 37 patients, occurring 4–6 months after repair of carotid endarterectomy (41). These events occurred at different hospitals over a 14-week period and were not caused by suture failure or infection. Histologic analysis demonstrated neovascularization and remodeling of the patches. As the authors suggested, it is possible that SIS-ECM patch strength is reduced during the remodeling process, which may increase risk for pseudoaneurysm in some cases.

Finally, SIS-ECM was used for patch angioplasty for femoral artery repair following endarterectomy in a case series of six patients (seven procedures) (42). Significant, early vascular complications occurred in three of the procedures (43%); patch rupture occurred in two procedures (29%), requiring immediate reoperation; and asymptomatic pseudoaneurysm occurred in one procedure, which was identified on routine follow up. No late complications were identified. Again, this case series used a four-ply SIS-ECM unlike the stronger six-ply SIS-ECM used in the present study.

Further, the lack of true pseudoaneurysm in the present report, including up to 24 months of follow up, suggest that this is a rare event. Additional long-term studies are required to better characterize this risk.

Infection of vascular patches is a potentially serious complication that may require removal of the patch and vein bypass to establish revascularization. Although patch infection may be underreported, studies have identified low rates of patch infection following endarterectomy repair (0–3%), with roughly similar rates across patch types (10, 15, 16, 29, 83, 84). In the histologic and observational data reported herein, no (0%) patch infections were identified. Resistance to infection may relate to the inherent antimicrobial properties of non-chemically crosslinked ECM (17, 49). In fact, SIS-ECM products have been successfully used in sites with active infection, whereas synthetic materials are generally avoided in the setting of infection (30, 58–60, 87–89).

In summary, non-chemically crosslinked ECM-based materials provide comparable clinical outcomes to other options for endarterectomy repair, but with several potential advantages (7, 16, 90). These materials, including SIS-ECM, release bioactive factors during early degradation following implantation, foster an immune response associated with constructive remodeling, provide sufficient mechanical strength and compliance similar to native arteries, resist infection, and, as demonstrated by the histology described above, remodel into site-appropriate vascular tissues. One advantage of the analyses presented above is that the data reflect real-world clinical practice and the performance of a 6-ply SIS-ECM patch in a heterogeneous, unselected patient population. Indeed, the outcomes were excellent, with high long-term patency rates, low incidence of adverse events, and compare favorably to outcomes of patch endarterectomy repair reported in the literature. It is possible that improved patient selection could further enhance the performance of this material in endarterectomy repair; future studies should seek to identify risk factors for adverse outcomes (e.g., restenosis) with SIS-ECM.

## Limitations

There are several limitations to the data presented above. First, the studies reported were not randomized or controlled trials; these data consist of real-world observational experiences that included only patients managed with SIS-ECM, thereby precluding the possibility of direct comparisons with other vascular patches. Second, follow up was limited, with some patients lost to follow up and a varying range of follow-up times. Longer and more complete follow-up will be required to confirm and extend the long-term safety and efficacy findings of SIS-ECM in iliofemoral and carotid endarterectomy. A more robust mechanism for ensuring follow-up may improve tracking of patients through long-term time points. Third, in the patients undergoing iliofemoral repair, the diagnosis of PAD was based on a comprehensive physical examination, and abnormal findings were confirmed by non-invasive diagnostic testing. However, results from these diagnostic tests were not collected as part of this dataset. There was a high incidence of patients with procedures performed for claudication in the iliofemoral group, yet documentation of the severity or progression of this symptom was not captured. Finally, the histologic analysis included a sample from only one patient.

The safety and efficacy findings reported in this manuscript conflict somewhat with previously published data that used SIS-ECM patch material for carotid or femoral endarterectomy patch repair. As outlined in the discussion, some authors reported similar success to that presented here but others have reported patch failures, early pseudoaneurysms, and/or restenosis. Because those data were generated from experiences at single centers, small patient populations, or four-ply versions of the SIS-ECM used in this report, this broader set of multi-center, multi-physician, six-ply ECM data can be expected to deviate from previous accounts. However, more robust studies are still needed to confirm the results observed herein, and larger randomized studies are needed to understand the full potential of SIS-ECM patches for vascular repair.

## Conclusion

Vascular patches play an essential role in the repair of arteriotomies following carotid and iliofemoral endarterectomy. The optimal choice of patch material remains unclear. Based on the perceived benefits of biologic materials, bovine pericardium has been widely used for this application, and recent clinical data support its efficacy and safety. In the present report, the clinical use of a biologic vascular patch material, which consists of six-ply SIS-ECM is reported. Observational data and histology from a complex case describe the reliable efficacy and strong safety of SIS-ECM for the repair of carotid and iliofemoral endarterectomies. The findings also demonstrate full tissue integration of the patch into endarterectomized vascular tissue, with low rates of restenosis, pseudoaneurysm, or other adverse vascular outcomes. Future research is needed to expand the understanding of the efficacy, safety, and host response to ECM-based materials in arterial reconstruction procedures.

## Data Availability Statement

The original contributions presented in the study are included in the article, further inquiries can be directed to the corresponding author.

## Ethics Statement

The studies involving human participants were reviewed and approved by either a Local IRB or Centralized IRB. The patients/participants provided their written informed consent to participate in this study.

## Author Contributions

KA, JA, HG, NM, SO, MP, and PS contributed substantially to the conception, design, data collection, and analysis of the work. SB contributed their expertise to the conception, design, analysis, and interpretation of data. All authors accept accountability for the accuracy of this work, and drafted, revised, and approved the final version of the manuscript to be published within this journal.

## Conflict of Interest

The authors declare that this study received funding from Aziyo Biologics, Inc. The funder had the following involvement with the study: study design, data collection and analysis, and supported preparation of the manuscript.
